# Major risk factors do not influence the outcomes of isolated medial patellofemoral ligament reconstruction in athletes with patellar instability: a prospective cohort study

**DOI:** 10.1186/s43019-026-00306-8

**Published:** 2026-03-03

**Authors:** Iván José Bitar, Bartolome Luis Allende, Lucas Daniel Marangoni, Damian Gabriel Bustos, Luciano Pezzutti, Lucia Belen Bitar, Juan Valentín Rivera Bacile

**Affiliations:** Sanatorio Allende, Avenida Hipólito Irigoyen 384, Nueva Córdoba, CP 5000, M85 L2 Causana, Malagueño, Córdoba, Argentina

**Keywords:** Medial patellofemoral ligament, Patellar instability, Risk factors, Return to sport, Clinical outcomes

## Abstract

**Background:**

Isolated medial patellofemoral ligament (MPFL) reconstruction is an effective, low-morbidity treatment for recurrent lateral patellofemoral instability (RLPI). The presence of major anatomical risk factors continues to generate controversy in surgical decision-making. It remains unclear whether the coexistence of multiple risk factors requires associated bony procedures or whether isolated MPFL reconstruction alone is sufficient. The purpose of this prospective study was to compare clinical outcomes, recurrence rates, return to sport, and patellofemoral degenerative changes in athletes with RLPI presenting with ≤ 1 versus 1 associated major risk factor treated with isolated MPFL reconstruction.

**Methods:**

This prospective cohort study included athletes aged 16–30 years with RLPI who underwent isolated MPFL reconstruction between 2013 and 2020. Patients were stratified according to the number of associated major risk factors (MRFs): ≤ 1 MRF (group 1) and 1 MRF (group 2). MRFs included trochlear dysplasia, patella alta, increased tibial tuberosity–trochlear groove distance, increased femoral anteversion, and increased tibial torsion. All patients underwent isolated MPFL reconstruction using the same surgical technique. Functional outcomes were assessed using the Knee injury and Osteoarthritis Outcome Score (KOOS), Kujala score, International Knee Documentation Committee (IKDC) score, and Lysholm score. Recurrence of instability, return to sport (RTS), and patellofemoral degenerative changes were evaluated. Minimum follow-up was 5 years.

**Results:**

A total of 86 athletes (43 per group) completed a minimum 5-year follow-up. Both groups showed significant postoperative improvements in all functional scores. At final follow-up, no between-group differences were observed (KOOS QoL mean difference 2.1; Kujala 0.8; IKDC 1.2; Lysholm 1.5; all *p* 0.05). MCID and PASS achievement rates were comparable between groups. Recurrence occurred in one patient per group (2.3%). More than 90% of athletes returned to sport at their preinjury level within 1 year. Mild, asymptomatic patellofemoral degenerative changes were observed in a small proportion of patients, with no between-group differences.

**Conclusions:**

With a medium-term follow-up, isolated MPFL reconstruction appeared to be a reliable and effective surgical option for treating RLPI in athletes from both groups. The procedure was associated with significant improvements in both primary and secondary outcomes and remained effective regardless of the number or type of associated major risk factors.

**Level of evidence:**

II, prospective cohort study.

## Introduction

After a first episode of lateral patellar dislocation, approximately one third of athletes will require surgical stabilization owing to recurrent lateral patellar instability [1]. Among surgical options, isolated MPFL reconstruction has gained popularity owing to its favorable outcomes and low morbidity [1–3]. The rationale for using isolated MPFL reconstruction as a standalone procedure lies in the critical role of the medial patellofemoral ligament as the primary restraint against lateral patellar translation [4]. Restoring its integrity effectively re-establishes patellar stability in many patients, potentially eliminating the need for more extensive and invasive procedures that target associated anatomical risk factors. However, surgical decision making is often influenced by the presence of major risk factors (MRFs), such as trochlear dysplasia, patellar height, patellar tilt, increased distance between the tibial tuberosity and trochlear groove (TT–TG), and femoral or tibial torsion, all of which are associated with higher recurrence rates [5]. There are two schools of thought advocating different surgical approaches. One school focuses on addressing as many associated MRFs as possible by performing “à la carte” surgical interventions [6]. According to this approach, if multiple MRFs are present, isolated MPFL reconstruction alone may be insufficient for long-term success, necessitating additional procedures such as derotational femoral or tibial osteotomy, medial tibial tubercle transfer, or even trochleoplasty. However, this strategy carries a potential risk of increased morbidity and surgical complications [5, 7]. In addition, literature still does not provide clear guidance on which MRFs should be prioritized, how many and what types of additional procedures are required, or the optimal thresholds for surgical intervention [8]. Regardless of the number of associated MRFs, the other school of thought recommends performing isolated MPFL reconstruction as a standardized surgery, given its low morbidity, low complication rates, low redislocation rates, and high patient satisfaction [9]. To our knowledge, this is the first prospective comparative study assessing the efficacy of isolated MPFL reconstruction according to the number of associated MRFs. The aim of this study was to compare functional outcomes, recurrence rates, return to sport, and patellofemoral degenerative changes in athletes with recurrent lateral patellar instability (RLPI) who had ≤ 1 MRF versus > 1 MRF and were treated with isolated MPFL reconstruction. The hypothesis was that no differences would be observed between the two groups.

## Methods

### Study design and patient selection

**Fig. 1:**
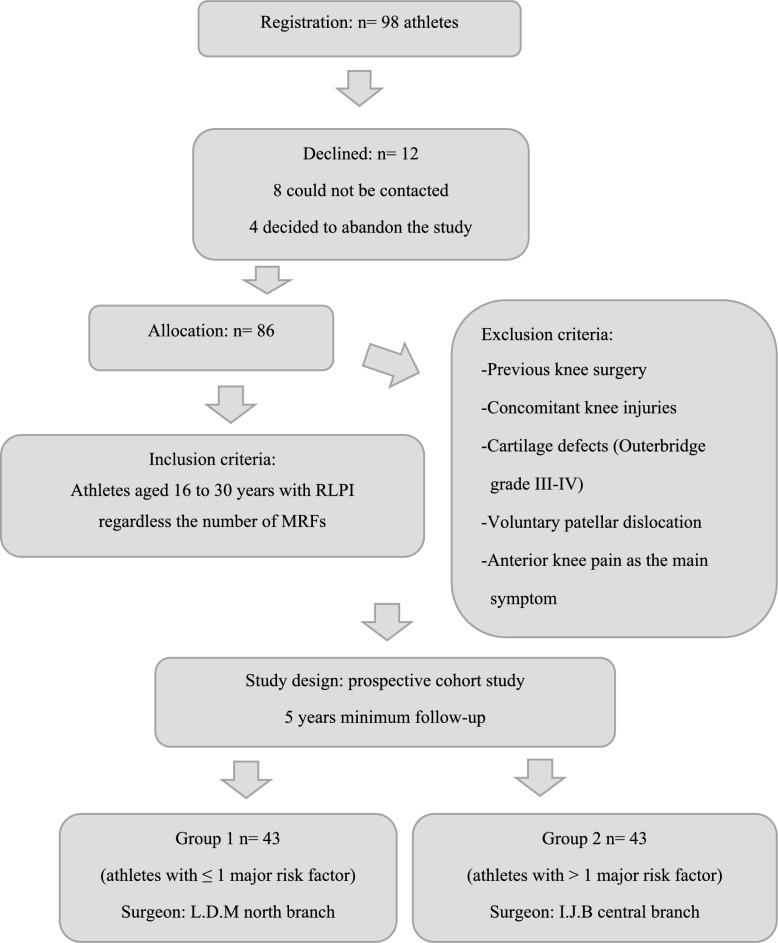
Methodological diagram, group conformation, and inclusion and exclusion criteria

With the approval of the ethics committee of our hospital (institutional review board (IRB): SA-2013-0513), a prospective cohort study involving 98 athletes with RLPI was conducted over an 8-year period (2013–2020). Informed consent was obtained from all patients. The inclusion criteria included athletes aged 16–30 years who had been diagnosed with RLPI, regardless of the type or number of associated major risk factors. Patients with generalized joint hyperlaxity were also included. The exclusion criteria were athletes with prior knee surgery or concomitant knee injuries (anterior cruciate ligament, collateral ligaments, fractures, or sequelae of previous trauma), severe patellar cartilage defects (Outerbridge grade III–IV), and pain as the primary symptom. RLPI was defined as the presence of two or more episodes of dislocation and/or subluxation. Hyperlaxity was defined as a Beighton score of ≥ 4 points. MRFs were defined as patella alta (Caton–Deschamps Index [CDI] > 1.2), TT–TG distance > 20 mm, trochlear dysplasia types A–D according to Dejour’s classification, tibial torsion > 30°, and femoral anteversion > 25° [10]. Regardless of the number and type of MFRs, group 1 consisted of athletes with ≤ 1 MFR, whereas group 2 consisted of athletes with > 1 associated MFR. Both groups were treated with isolated MPFL reconstruction as surgical treatment. The allocation to each group was determined by the hospital branch where the patients were treated. All surgeries were performed by two experienced knee surgeons, each with over 10 years of experience. One surgeon (L.D.M.) operated on group 1 at the northern branch of our hospital, whereas the other surgeon (I.J.B.) operated on group 2 at the central branch (Fig. 1).

### Surgical technique and rehabilitation

**Fig. 2:**
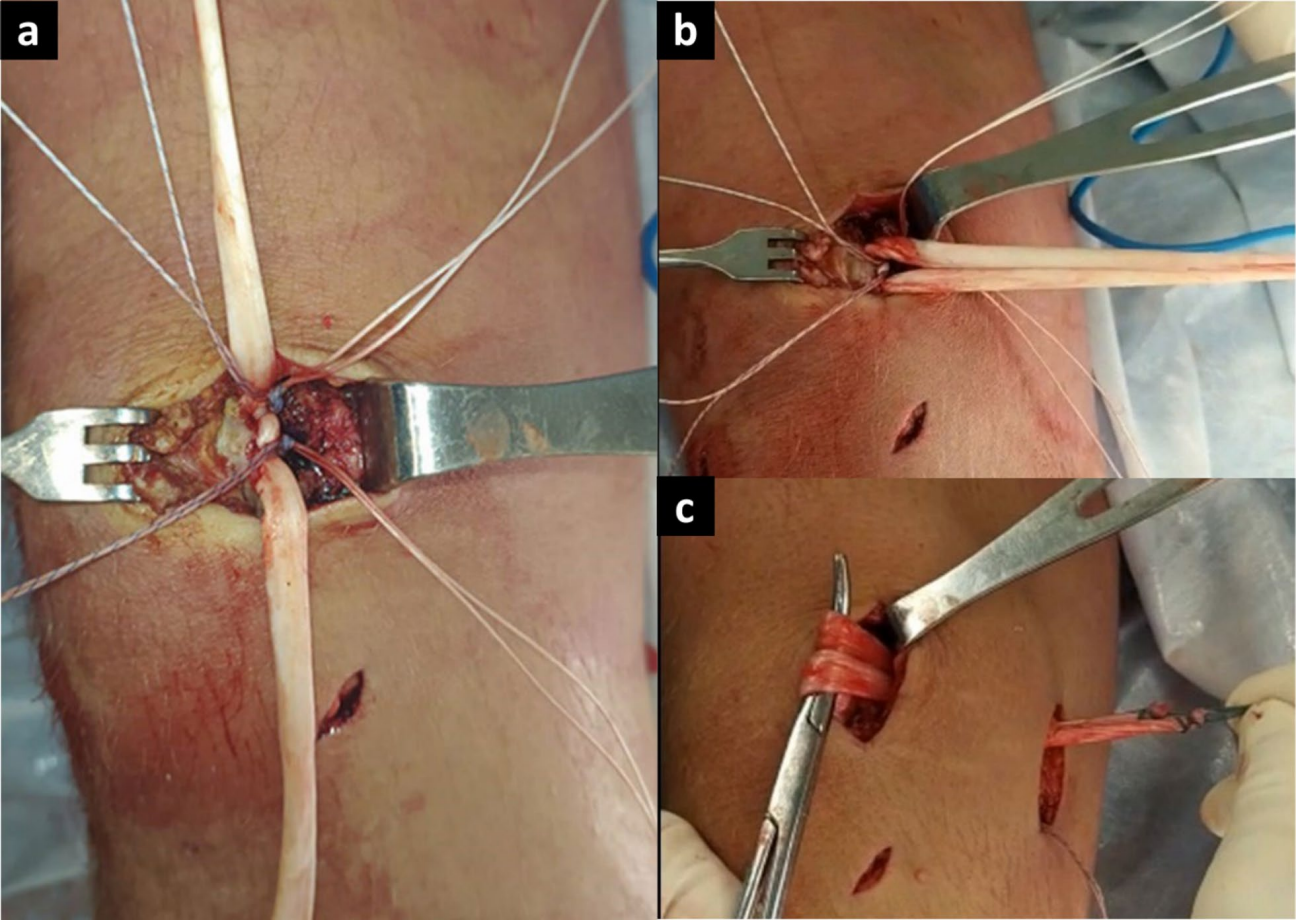
**a** Exposure of the medial and proximal patellar borders. Two 5.5-mm PEEK double-loaded anchors placed in the patellar groove to fix the graft. **b** Semitendinosus graft prepared for passage. **c** Graft passed between deep joint capsule (layer 3) and vastus medialis aponeurosis (layer 2)

**Fig. 3:**
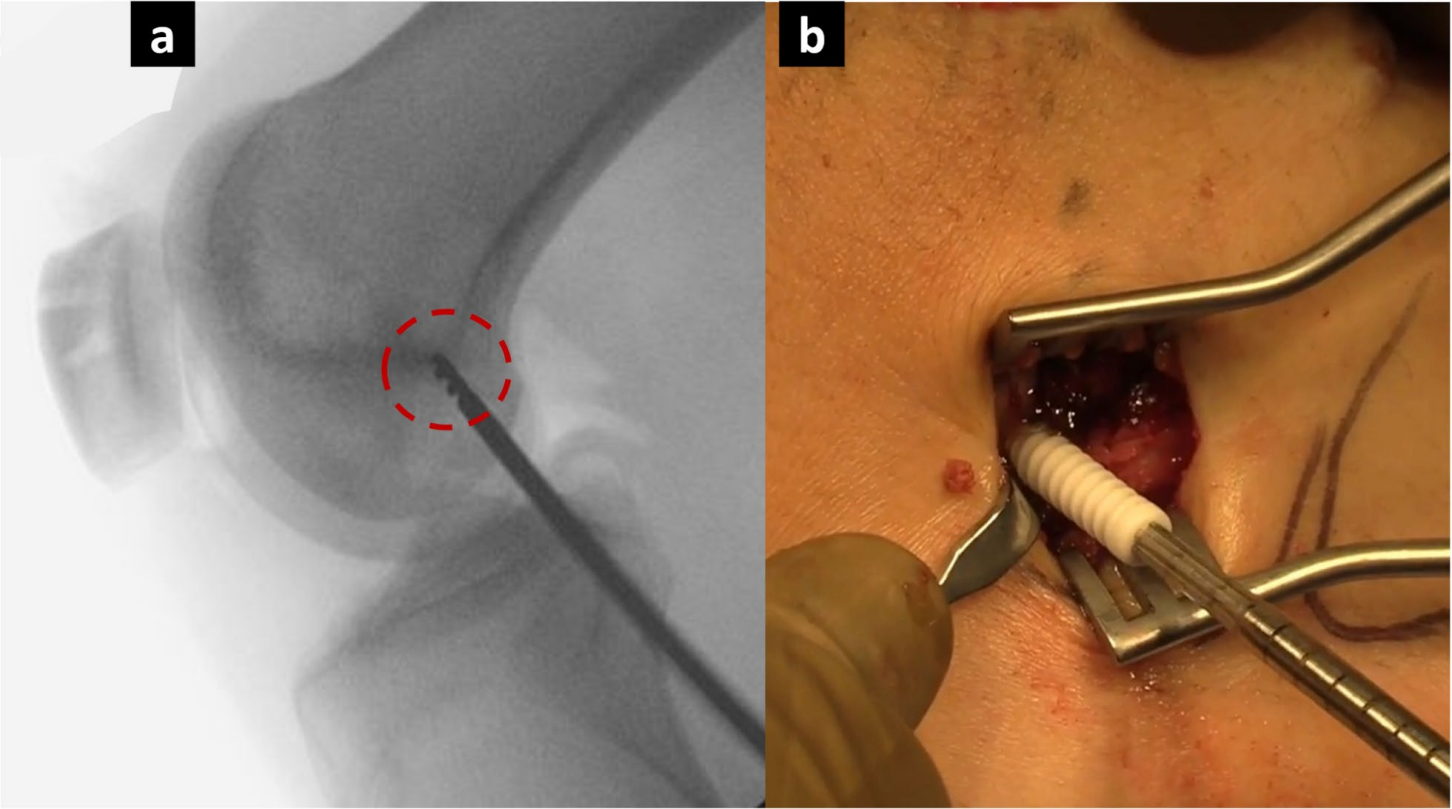
**a** Schöttle’s point identified under fluoroscopic guidance. **b** Graft fixation with a PEEK tenodesis screw at 60° of knee flexion

Patients were placed in the supine position, and spinal anesthesia was administered in all patients. A nonsterile tourniquet was applied to the upper thigh. Diagnostic knee arthroscopy was performed to assess the patellar and trochlear chondral surfaces, remove loose bodies, and thoroughly examine the knee joint. The semitendinosus tendon was harvested through a 2-cm incision centered over the pes anserina insertion via an open-ended tendon stripper. The graft was trimmed of any muscle tissue, and a mark was placed at its midpoint. Next, a 3-cm incision was made in the proximal third along the medial border of the patella. Dissection between the retinaculum and the deep joint capsule was carried out via Metzenbaum scissors, with care taken to avoid damage to the articular cartilage. The middle and proximal thirds of the patella were exposed via electrocautery. A bone groove was created in the upper third and the proximal part of the middle third of the patella via a rongeur. Two 5.5 mm polyetheretherketone (PEEK) anchors (Fergus, Promedon) with double sutures were placed 1 cm apart in the bone bed. The graft was then secured to the patella, with its midpoint positioned between the anchors, and the sutures were tied (Fig. 2). A 2–3-cm incision was made at the femoral level over the groove of the medial patellofemoral ligament (MPFL), located between the femoral epicondyle and the adductor tubercle. A guide wire was inserted slightly proximal and posterior to the medial epicondyle and slightly anterior and distal to the adductor tubercle (Schottle’s point [11]) (Fig. 3). Pin placement was confirmed using fluoroscopy, and a drill was used to create a bone tunnel measuring 6–8 mm, depending on the graft. The graft was then passed between the previously dissected layers. With the knee at 60° of flexion, the graft ends were pulled through the tunnel and secured via a PEEK tenodesis screw (Fergus, Promedon) [12]. Flexion and extension movements were performed to confirm the stability of the graft fixation and the correct patellar tracking through a standard anterolateral portal. The same rehabilitation protocol was applied in both groups. During the first 4 weeks, patients were allowed to ambulate with partial weight-bearing via crutches and the use of a protective orthopedic brace. A range of motion (ROM) from 0° to 90° was permitted from the first postoperative day. After the initial 4 weeks, the brace was removed, full weight-bearing was allowed, and the rehabilitation program was continued.

### Functional and imaging assessment

In this study, data were collected preoperatively and at 6, 12, and at least 60 months postoperatively. Primary outcomes were assessed using the Knee Injury and Osteoarthritis Outcome Score (KOOS), the Kujala score, the International Knee Documentation Committee (IKDC) score, and the Lysholm score [13, 14]. The recurrence rate was also considered a primary outcome. The percentage of patients who exceeded the minimal clinically important difference (MCID) and the patients acceptable satisfactory state (PASS) for the KOOS-quality of life (QoL), Kujala, IKDC, and Lysholm scores were evaluated in each group. The MCIDs for the KOOS-QoL, Kujala IKDC, and Lysholm scores were 12.7, 9.1, 9.9, and 11.1, respectively, whereas the PASSs for the KOOS-QOL, Kujala, IKDC, and Lysholm scores were 53.1, 85.5, 73.2, and 75.5, respectively [13, 15]. Postoperative data on return to sport (RTS) and patellofemoral degenerative changes were also collected as secondary outcomes. We categorized RTS into four grades: grade 1, return to the same sport at the same level; grade 2, return to the same sport at a lower level; grade 3, discontinuation of the preinjury sport with a change to a different sport; and grade 4, cessation of all sports activities. Preoperative imaging was used to diagnose associated MRFs. A lateral radiograph at 30° of knee flexion was used to detect patella alta through the Caton–Deschamps method, whereas magnetic resonance imaging (MRI) was performed to confirm preexisting patellofemoral osteoarthritis, assess MPFL tears, and measure the TT–TG distance [16, 17]. Lateral and axial X-rays were utilized to classify trochlear dysplasia according to Dejour’s classification, whereas computed tomography (CT) scan imaging was performed to assess femoral and tibial torsion in all patients [16, 18, 19]. At the end of the follow-up period, lateral and axial X-ray images at 30° of knee flexion were obtained from all patients to assess degenerative changes in the patellofemoral joint via the Kellgren–Lawrence classification system [20]. All preoperative and postoperative images were assessed by a single knee imaging specialist. Evaluations unrelated to imaging were conducted by the same surgeons and two sports medicine fellows. As the fellowship program is 1-year long, multiple fellows contributed to the analysis of the data.

## Statistical analysis

An a priori power analysis was performed to determine the required sample size to detect a clinically relevant difference in RLPI between the two groups. On the basis of previous literature and institutional data, we assumed an expected RLPI rate of 10% in both groups and aimed to detect a 20% absolute difference, which was considered clinically meaningful. Assuming a two-tailed test with a significance level (*α*) of 0.05 and a power (1 − *β*) of 0.80, the estimated minimum sample size required was 40 patients per group. We also anticipated that 90% of recurrences would occur within the first 24 months of follow-up. For the analysis, categorical variables were expressed as frequencies and percentages and compared using the chi-squared test or Fisher’s exact test. Continuous variables were expressed as mean (standard deviation, SD) or median (interquartile range, IQR), as appropriate, and compared using the independent *t*-test or Mann–Whitney *U* test. Between-group differences were reported with 95% confidence intervals in addition to p-values. The Shapiro–Wilk test was applied to assess normality. A *p*-value < 0.05 was considered statistically significant. Data was analyzed with SPSS for Windows 7, version 18.0 (SPSS Inc., Chicago, IL, USA).

## Results

### Baseline demographics and prevalence of major risk factor.

Among the 98 athletes initially enrolled, 86 (43 in each group) completed the minimum 5-year follow-up. Two groups of 43 patients were ultimately analyzed, with no significant differences in baseline demographics (Table 1). Trochlear dysplasia was the most common MRF, present in 48.8% of group 1 and 65.1% of group 2. Patella alta was present in 30% of Group 1 and 46.5% of Group 2 (Table 2).

**Table 1:** Baseline demographic characteristics of the included patients

Variables		Group 1	Group 2	*p *value
Patients (no.)	86	43	43	–
Sex* (no.)				
Male/female		25/18	20/23	0.26
Average age (min–max)		19.5 (17–30)	22.7 (14–25)	0.63
Time between surgery/first injury (*y*)		3.8 (1–3)	2.8 (1–4)	0.81
Side (right/left)		28/15	24/19	0.46
Follow up (months)		68.3 (60–120)	72.3 (60–110)	0.21
Type of sport				
– Collision				
Rugby		8	3	
– Contact				
Soccer		14	18	
Basketball		6	4	
Volleyball		4	5	
Handball		3	1	
Hockey		1	1	
Judo		1	–	
– Non-contact				
Cross-fit		1	3	
Paddle		1	6	
Running		1	–	
Tennis		1	1	
Cross-country		1	1	
Spinning		1	–	

^*^The results between the two groups remained coherent after independent analysis

**Table 2:** Frequency of major risk factors

Major risk factor	Total frequency	Group 1	Group 2
Trochlear dysplasia A	5	3 (6.9%)	2 (4.6%)
Trochlear dysplasia B	32	14 (32.5%)	18 (41.8%)
Trochlear dysplasia D	7	1 (2.3%)	6 (13.9%)
Trochlear dysplasia C	5	3 (6.9%)	2 (4.6%)
Patella Alta	29	13 (30.2%)	20 (46.5%)
Increased TT–TG distance	25	5 (11.6%)	20 (46.5)
Increased tibial torsion	11	1 (2.3%)	10 (23.2%)
Increased femoral anteversion	8	1 (2.3%)	7 (16.2%)
No major risk factor	–	2 (4.6%)	0

### Primary outcomes

The mean KOOS QoL, Kujala, IKDC, and Lysholm scores significantly improved from preoperative to postoperative assessments in both groups (Table 3). At final follow-up, no significant between-group differences were observed (KOOS QoL: mean difference = 2.1; 95% CI −1.5 to 5.7; Kujala: mean difference = 0.8; 95% CI −3.6 to 5.1; IKDC: mean difference = 1.2; 95% CI −4.4 to 6.8; Lysholm: mean difference = 1.5; 95% CI −2.7 to 5.7) (Table 4). The rates of achieving MCID and PASS thresholds were high and comparable between groups (MCID: KOOS QoL 93% versus 95%, Kujala 100% versus 96%, IKDC 83% versus 81%, Lysholm 76% versus 75%; PASS: KOOS QoL 91% versus 88%, Kujala 76% versus 73%, IKDC 86% versus 88%, Lysholm 78% versus 80%; all *p* > 0.05). Recurrence occurred in one patient (2.3%) in each group. Both soccer players were successfully managed with rehabilitation and tibial tubercle osteotomy (TTO). Additionally, two group 2 athletes demonstrated positive apprehension at12 months. One patient presented with Dejour type B trochlear dysplasia, patella alta, and increased tibial torsion, whereas the other had Dejour type D trochlear dysplasia with an increased TT–TG distance. Both also exhibited muscle hypotrophy, which resolved with strengthening exercises.

**Table 3:** Outcomes of KOOS-QoL score, Kujala score, IKDC score and Lysholm score (media—standard deviation). Preoperative and postoperative comparison of each group

Score	Group 1		*P*-Value	Group 2		*p*-Value
	Baseline	> 5 years		Baseline	> 5 years	
KOOS-QoL	38.3 ± 12.3	88.3 ± 12.3	< 0.0001	36.2 ± 9.5	86.3 ± 6.4	< 0.0001
Kujala	64.2 ± 16.1	90.2 ± 11.7	< 0.0001	66.3 ± 20.3	91.2 ± 8.7	< 0.0001
IKDC 53.2 ± 13.2		84.6 ± 11.1 < 0.0001		54.6 ± 9.2 85.3 ± 10.5		< 0.0001
Lysholm 62.6 ± 12.1		94.4 ± 9.5 < 0.0001		66.2 ± 10.2 92.8 ± 8.9		< 0.0001

KOOS-QoL, Knee injury and Osteoarthritis Outcome Score–Quality of Life; IKDC, International Knee Documentation Committee

**Table 4:** Outcomes of KOOS-QoL score, Kujala score, IKDC score, and Lysholm score at a mean follow-up of 5 years

Score	Group 1	Group 2	*p*-Value
KOOS-QoL	88.3 ± 12.3	86.3 ± 6.4	0.19
Kujala	90.2 ± 11.7	91.2 ± 8.7	0.73
IKDC	84.6 ± 11.1	85.3 ± 10.5	0.68
Lysholm	94.4 ± 9.5	92.8 ± 10.5	0.46

Comparison between groups

KOOS-QoL, Knee injury and Osteoarthritis Outcome Score–Quality of Life; IKDC, International Knee Documentation Committee

### Secondary outcomes

More than 90% of athletes in both groups achieved RTS grade 1 (return to the same sport at the same level) within 1 year (group 1: 91%; group 2: 93%; difference = −2%; 95% CI −12% to 8%) (Table 5). Mild patellofemoral degenerative changes (Kellgren–Lawrence grade 1) were observed in 2 patients (4.6%) from group 1 and 3 patients (6.9%) from group 2 (difference = −2.3%; 95% CI −11% to 6%; *p* = 0.53). None of the affected athletes reported pain, and no surgery-related complications were noted.

**Table 5:** Return to sporting level at end of follow-up at a mean follow-up of 5 years

Grade	Group 1	Group 2	*p*-Value
1	15 (34.8%)	17 (39.5%)	0.71
2	26 (60.4%)	25 (58.1%)	0.36
3	2 (4.6%)	1 (2.3%)	0.47
4	0	0	–

Comparison between groups

Grade 1, return to the same sport at the same level; Grade 2, return to the same sport at a lower level; Grade 3, cessation of the preinjury sport (change of sport); Grade 4, cessation of sports activity

## Discussion

The present study demonstrated that isolated MPFL reconstruction provides satisfactory functional outcomes, low recurrence rates, and high return-to-sport rates in athletes with RLPI, regardless of the number or type of associated major risk factors. These findings support isolated MPFL reconstruction as a reliable option for restoring patellar stability in appropriately selected athletes, offering low morbidity and fewer complications compared with combined procedures. Importantly, these results should not be interpreted as dismissing the role of bony realignment procedures, which remain indicated in cases with severe anatomical abnormalities [21].

Our findings are consistent with previous reports demonstrating favorable outcomes after isolated MPFL reconstruction, even in the presence of predisposing anatomical factors [9, 14]. Erickson et al. and Lee et al. showed significant improvements in KOOS, Kujala, IKDC, and Lysholm scores regardless of trochlear dysplasia, patellar height, or increased TT–TG distance [1, 22]. Other authors have also reported comparable patient-reported outcome measures (PROMs) outcomes between isolated MPFL reconstruction and combined procedures such as TTO or trochleoplasty [21, 23]. However, adding bony procedures increases operative time, morbidity, and complication rates [24, 25]. A recent meta-analysis reported a 10.1% complication rate for MPFL reconstruction combined with TTO versus 5.4% for isolated reconstruction, with higher rates of tibial fractures, hardware irritation, and delayed RTS [24]. Our recurrence rate is consistent with previously reported outcomes for both isolated and combined MPFL reconstructions, in which recurrence remains low (≤ 6%) [1, 21, 26]. In contrast, complication rates tend to rise when bony procedures are added [21, 24]. Lehane et al. reported a 27.5% complication rate and a 16.6% reoperation rate after TTO, while van Sambeeck et al. described 190 complications in 822 knees treated with trochleoplasty (~23%) [27, 28]. Similarly, DeNovio et al. reported recurrence rates of 3–8% with higher complication (17%) and reoperation (16%) rates [29]. These findings indicate that, although combined procedures may improve stability in selected cases, they do so at the expense of greater postoperative morbidity, highlighting the importance of careful patient selection and a balanced risk–benefit assessment.

In our study, group 2 consisted of athletes with two or more anatomical risk factors, whose threshold values have been previously described—CDI > 1.2, TT–TG > 20 mm, trochlear dysplasia, femoral anteversion > 25°, and tibial torsion > 30° [4, 5]. However, there are still no evidence-based clinical guidelines clearly defining the threshold at which an osteotomy should be indicated or which MRF should be prioritized to optimize outcomes [30]. The favorable results observed in this group raise the question of when a combined procedure is truly necessary. In our cohort, 90% of athletes in both groups returned to sport within the first year, a finding that may reflect the lower morbidity associated with isolated MPFL reconstruction compared with techniques involving osteotomy. In this regard, Krych et al. reported slower RTS and persistent quadriceps weakness at 6 months after MPFL reconstruction combined with TTO [31], and Lampros et al. noted that only 58% of patients achieved symmetric quadriceps strength at 6 months even after isolated MPFL reconstruction, suggesting that adding an osteotomy may further delay early functional recovery [32].

Previous studies have suggested that performing isolated MPFL reconstruction without correcting certain associated MRFs could contribute to the development of long-term patellofemoral degenerative changes [33, 34]. However, other authors have reported conflicting findings [35, 36]. In a long-term retrospective study, Hashimoto et al. found no progression of patellofemoral osteoarthritis in patients treated with MPFL reconstruction alone or in combination with TTO [37]. Similarly, Shatrov et al. reported that, at an average follow-up of 12 years, only one third of 54 patients with RLPI treated with isolated MPFL reconstruction developed Iwano stage 1 or 2 patellofemoral osteoarthritis [38]. Consistent with these results, and with a mean follow-up of 68 months for group 1 and 72 months for group 2, our study observed only minimal patellofemoral degenerative changes, limited to three asymptomatic patients.

This study has several limitations that should be acknowledged. First, randomization was not possible owing to healthcare system–related factors influencing patient assignment. Second, although both surgeons were experienced and followed the same technique and rehabilitation protocol, subtle differences in surgical practice or perioperative care may have influenced the outcomes. Third, 12 patients were lost to follow-up, and the duration of follow-up was insufficient to fully assess long-term results. Fourth, data collection by multiple fellows over the years may have introduced variability. Fifth, the inclusion of athletes from different sports and competition levels limited sample uniformity. Finally, the absence of a control group treated with combined MPFL reconstruction and bony procedures restricts direct comparisons with more extensive surgical approaches.

## Conclusions

With a medium-term follow-up, isolated MPFL reconstruction appeared to be a reliable and effective surgical option for treating RLPI in athletes from both groups. The procedure was associated with significant improvements in both primary and secondary outcomes and remained effective regardless of the number or type of associated major risk factors.

## Data Availability

All data are available under reasonable request to the corresponding author.

## Abbreviations

RLPI: Recurrent lateral patellar instability
MRFs: Major risk factors
KOOS: Knee Injury and Osteoarthritis Outcome Score
IKDC: International Knee Documentation Committee
TT–TG: Tuberosity–trochlear groove
CDI: Caton–Deschamps index
MPFL: Medial patellofemoral ligament
ROM: Range of motion
RTS: Return to the same sport
MRI: Magnetic resonance imaging
TTO: Tibial tubercle osteotomy
